# Spatial Cognition in Teleost Fish: Strategies and Mechanisms

**DOI:** 10.3390/ani11082271

**Published:** 2021-07-31

**Authors:** Fernando Rodríguez, Blanca Quintero, Lucas Amores, David Madrid, Carmen Salas-Peña, Cosme Salas

**Affiliations:** Laboratorio de Psicobiología, Universidad de Sevilla, 41018 Sevilla, Spain; fernanr@us.es (F.R.); bqvera@us.es (B.Q.); lucas.amores.carrera@gmail.com (L.A.); dmadrid@us.es (D.M.); carmensp224@gmail.com (C.S.-P.)

**Keywords:** teleost fish, spatial navigation, spatial strategies, telencephalon, optic tectum, hippocampal pallium, vertebrate brain evolution

## Abstract

**Simple Summary:**

The study of the neurobiological basis of spatial cognition has been demonstrated to be one of the most exciting, successful, and productive research fields in neuroscience. An enormous number of experimental results on the brain mechanisms of navigation have been obtained over several decades and a number of theories and detailed mechanistic computational models have been developed to account for these data obtained mainly in mammals and birds. Recently, the use of teleost fish species as animal models in neurobiology has exponentially increased, nicely complementing the use of traditional mammalian models in basic and translational neuroscience research. Comparative neurobiological research has shown that teleost fish can use a variety of navigational strategies that closely resemble those described in mammals and birds. Although some of these similarities could indicate evolutionary convergence shaped by common environmental constraints and survival requirements, at least some of these strategies seem to be based on conserved neural substrata likely shared with land vertebrates, suggesting that these strategies and their neurobiological basis could have appeared very early on during vertebrate evolution.

**Abstract:**

Teleost fish have been traditionally considered primitive vertebrates compared to mammals and birds in regard to brain complexity and behavioral functions. However, an increasing amount of evidence suggests that teleosts show advanced cognitive capabilities including spatial navigation skills that parallel those of land vertebrates. Teleost fish rely on a multiplicity of sensory cues and can use a variety of spatial strategies for navigation, ranging from relatively simple body-centered orientation responses to allocentric or “external world-centered” navigation, likely based on map-like relational memory representations of the environment. These distinct spatial strategies are based on separate brain mechanisms. For example, a crucial brain center for egocentric orientation in teleost fish is the optic tectum, which can be considered an essential hub in a wider brain network responsible for the generation of egocentrically referenced actions in space. In contrast, other brain centers, such as the dorsolateral telencephalic pallium of teleost fish, considered homologue to the hippocampal pallium of land vertebrates, seem to be crucial for allocentric navigation based on map-like spatial memory. Such hypothetical relational memory representations endow fish’s spatial behavior with considerable navigational flexibility, allowing them, for example, to perform shortcuts and detours.

Most studies on spatial cognition have conventionally focused on mammals and birds, which are usually considered “advanced” vertebrates that supposedly share with humans a complex behavioral repertoire based on higher forms of perceptual, cognitive, and emotional functions, as well as increased learning and memory capabilities and executive guidance. In contrast, other groups of vertebrates such as teleost fishes, traditionally regarded as more primitive or “less advanced”, have been studied to a much lesser extent. This absence of information has reinforced the misleading view that the behavior of these “lower” vertebrates relies on more simple mechanisms, mainly on fixed action patterns and unlearned predispositions, with learning and memory playing a limited role in fish behavior. However, an increasing amount of empirical evidence indicates that the behavioral and cognitive capabilities of teleost fish, as well as the complexity of their brains, have been frequently underestimated. Teleost fish, the largest clade of fishes belonging the class Actinopterygii, or ray-finned fishes, are an extremely successful zoological group that represent almost half of all the vertebrate species combined. They occupy an enormous variety of aquatic habitats and display an amazing diversity of morphological and functional adaptations. Notwithstanding, they also share some brain and behavioral characteristics with other vertebrates. Teleost fish exhibit sophisticated spatial orientation and navigation skills, which are essential for survival and reproduction in nature. Teleosts need to navigate through their environments to find food, avoid predation, and return to their homing territories. Having the capability to find their way through an environment and remembering the place of certain events or the precise location of some objects can be beneficial for fish survival and reproduction success. These spatial cognition capabilities are based on specialized brain mechanisms that underlie the processes of perception, learning, memory, planning, and behavioral output required for navigation. In particular, teleost fish seem to be able to use advanced spatial navigation strategies that parallel those of land vertebrates, including mammals.

## 1. Spatial Cognition in Teleost Fish

Early naturalistic studies described a rich spatial behavior repertoire in teleost fish and suggested that at least some of these skills could be based on complex learning and memory mechanisms [1,2]. Fish move very effectively over a wide range of geographic scales, from small trips through their usual territories of residence to intercontinental migrations. Many teleost fish species are sedentary and territorial, remaining attached to a particular home range for foraging and reproduction which guards them from competitors [3,4,5,6], and are able to return to their territories after being artificially displaced kilometers away from their usual residence area, even after several months of absence [3,7,8,9,10,11]. In addition, other teleost species are able to successfully undertake trans-oceanic journeys [12,13,14]. Both territorial attachment and journeys require well-developed spatial cognition capabilities, such as recognizing and remembering landmarks and a terrain’s spatial structure, and the capability of using local and directional information combined with these memories to orient and navigate through the environment.

### 1.1. Teleost Fish Can Use Multiple, Parallel Spatial Strategies for Navigation

Fish can rely on a multiplicity of sensory cues and sources of spatial information for orientation and navigation. For example, they can use visual [15,16,17,18], olfactory [19,20], auditory [21,22], lateral line [23,24,25], and electrosensory information [26,27,28,29], as well as diverse sources of directional information to orient and navigate, such as sun position [30,31], polarized light gradient [32,33], geomagnetic compass [34,35], or water current direction [36,37]. In addition, like mammals and birds, teleost fish can use a variety of spatial navigation strategies that are dissociable at behavioral and neural levels. Some authors have categorized in different hierarchies the variety of navigation strategies that animals can potentially use. For example, it has been proposed that spatial strategies can range from taxis, stereotyped stimulus-response associations, and guidance behavior, based on egocentric (“body-centered”) frames of spatial reference, to allocentric (“external world-centered”) navigation based on “cognitive maps” of the environment [38,39]. Other classifications separate “local navigation” strategies (i.e., target or beacon approaching, snapshot orienting, recognition-triggered responses, path-integration, route following), based on current sensory information provided by the immediately perceivable environment, from “way-finding” strategies (i.e., topological navigation, survey or metric navigation), that involve the use of some sort of spatial representation of environmental information about terrains placed beyond the current range of perception [40,41,42,43,44,45,46,47,48,49,50,51]. Carefully controlled laboratory experiments have shown that fish can generate egocentrically referenced orientation responses, centered on the animal’s receptive surfaces or body axes, such as turning at a determined angle at the choice point in a plus-maze or guidance by local visual cues or a beacon associated with the goal position. These experiments also showed that, in addition to egocentric spatial strategies, fish can perform “place” responses, potentially denoting the use of an allocentric (“world-centered”) spatial coordinate reference system for navigation, likely based on map-like memory representations anchored to the spatial environment and independent of the subject’s own position [17,52,53].

**Figure 1:**
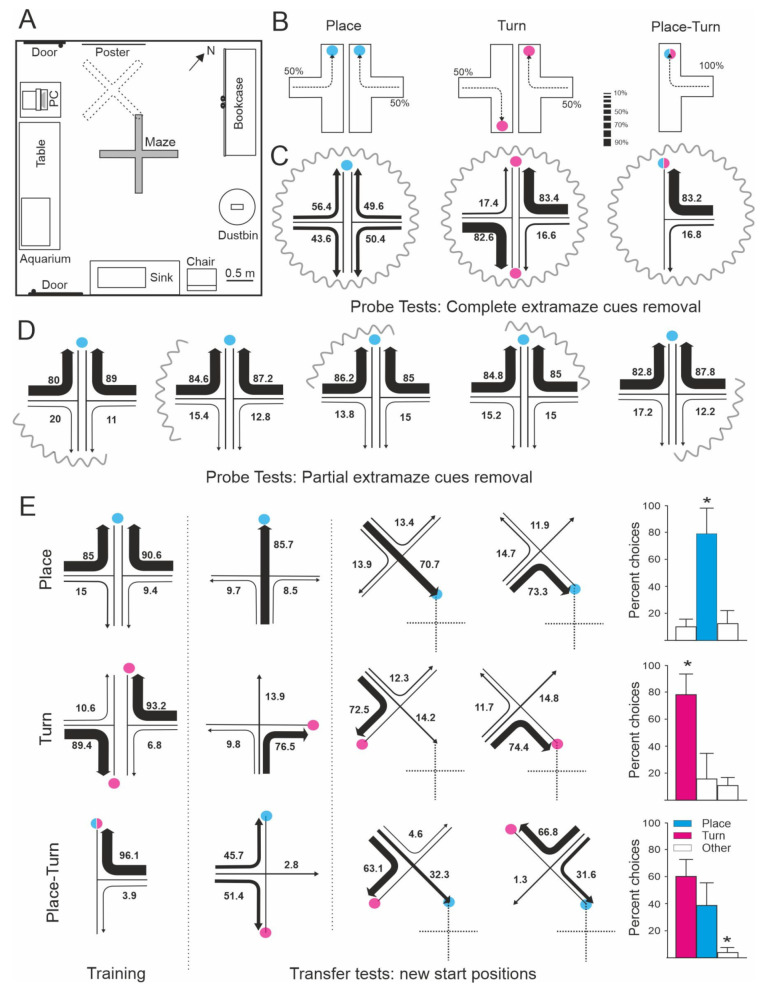
Spatial navigation strategies used by goldfish to solve different procedures in a four-arm maze. (**A**) Experimental room showing the maze in its training position (solid line) and in its rotated and displaced position used in the transfer tests (dotted line), and the extramaze cues. (**B**) Training procedures. Arrows show the most effective path to reach the goal. *Place* and *turn* procedures used two different start positions randomly assigned across trials (50% each). The colored circle marks the goal location in each procedure. (**C**) Percentage of choices in the probe test in which all the extramaze cues were occluded by means of curtains. The numbers and the relative thickness of the arrows denote the percentage of times that a particular choice was made. (**D**) Percentage of choices by the animals in the *place* procedure in the probe tests in which only a part of the extramaze cues were occluded. (**E**) Trajectories chosen by the animals in the different groups during training and transfer trials in which new start positions were employed. In one type of transfer tests (left) the maze remained in its usual position, in the other type (right), the maze was displaced in the room in such a way that the end of one arm was located in the same place where the fish were rewarded during training trials. The dashed lines indicate the original position of the maze during training. The blue circles mark the goal place for animals in the *place* and the *place-turn procedures*. The red circles mark the goal for the turn group during training and the arm corresponding with an egocentric (turn) strategy for both the *turn* and the *place-turn* procedures. The histograms on the right show the accumulated mean percentage of choices during the transfer tests. Asterisks denote significant differences. Modified from [17].

An experimental demonstration showing that teleost fish can use a variety of spatial strategies for navigation was provided by Rodríguez et al. [17] (Figure 1). In this study, goldfish were trained to solve different tasks in a four-arm maze placed into a spacious room with plenty of visual cues. In one of these tasks the animals had to perform a fixed turn response at the choice point in the maze irrespective of the arm of departure (*turn* procedure). A second group of goldfish were trained to reach the arm of the maze coinciding with a particular place in the room defined by an array of extramaze cues, with the turn direction irrelevant to the solving of the task (*place* procedure). A third group of animals were trained in a mixed procedure in which the goal could be reached using both a simple turn response or/and a place response based on the extramaze cues (*turn-place* procedure). Although the animals in all procedures readily learned to reach the goal with accuracy, the subsequent transfer and probe tests revealed that they were using very different spatial strategies. During transfer tests in which novel departure positions were used, the animals in the *turn* procedure chose predominantly the arm that coincided with the 90° turn response learned in the training trials, and this behavior was not altered during the probe tests in which the extramaze cues were curtained off (Figure 1C,E). These results denoted that the animals trained in the *turn* procedure learned to rely on a purely idiothetic, body-centered reference system to find the baited feeder, and that these animals did not take into account allothetic information for task solution. In contrast, during the transfer tests in which they were forced to depart from novel start positions, the goldfish in the *place* procedure preferentially swam to the previously rewarded goal location (place response), irrespective of turn direction, and they were lost during the probe tests in which the extramaze visual cues were deleted, indicating that they solved the task using a “place” strategy based on allothetic information (Figure 1C–E). Moreover, the results of the transfer and probe tests demonstrated that the goldfish trained in the mixed *turn-place* procedure used turn and place strategies concurrently, and that they switched from one to another depending on the task requirements and the kind of information available (Figure 1C,E). The cooperative use of separate, but complementary, spatial strategies could explain the better performance observed in the animals in the *turn-place* procedure compared to the animals in the other groups that likely used one of these strategies alone. Additional experiments provided further and converging evidence about the use of multiple, parallel spatial cognition strategies for orientation and navigation in teleost fish. For example, López et al. [16,54] showed that goldfish can use cue (i.e., egocentric guidance) and place (allocentric) strategies cooperatively, or shift flexibly between them. Usually the environment provides multiple and redundant sources of spatial information, thus the parallel operation of different spatial strategies could increase navigational efficiency and diminish the occurrence of flawed or inaccurate responses [17,55,56,57,58,59,60,61,62,63,64,65,66,67,68]. Interestingly, as discussed below, brain lesion experiments showed that these different strategies are based on separate memory systems that can also be dissociated on the basis of their neural substrata. For example, telencephalon ablation selectively impairs place strategies in goldfish, sparing, or even having beneficial effects, on the use of turn or guidance strategies [54,69,70,71].

### 1.2. Map-Like Memories and Fish Navigation

Therefore, a considerable amount of naturalistic and experimental evidence indicates that teleost fish rely on multiple sources of spatial information and can use sophisticated navigational strategies. Some of these spatial strategies necessarily involve complex learning and memory capabilities and require the concurrence of flexible representational mechanisms, the most remarkable of which probably is allocentric navigation based on map-like spatial memory [38,39,42,51,53,72,73,74]. Such hypothetical memory representations presumably encode the spatial relationships between all the known places and landmarks in a common allocentric reference frame or unitary global map, accessible as a whole, and that can operate to infer the spatial relationships between any of the represented elements. Therefore, these internal representations likely allow animals to accurately and flexibly navigate within the environment, for example, using incomplete patches of spatial information, or planning optimal trajectories to any intended target location, even when departing from unfamiliar places implies traversing unknown terrains to the goal.

Allocentric (“world-centered”) navigation based on cognitive maps or survey representations is considered the highest and most elaborated mechanism in the hierarchy of navigational strategies, and commonly supposed as a capability owned exclusively by birds and mammals. Notably, several laboratory studies using behavioral procedures comparable to those typically used to test spatial memory in mammals have reported map-like spatial memory-based navigation abilities in teleost fish. For example, in the above described experiment by Rodríguez et al. [17] the goldfish trained to navigate to a fixed location in a four-arm maze surrounded by an array of distal visual cues (place task) were disoriented during the probe tests in which the maze was completely encircled by curtains, evidencing that they relied on the extramaze visual cues. However, these goldfish still were able to successfully find the goal when the extramaze cues were partially occluded, which imply that the goal place was defined by its redundant spatial relationships with multiple cues. Similar results were reported by Durán et al. [69], showing that goldfish can locate a particular feeder in a matrix of 25 feeders, maintaining invariable spatial relationships within an array of distributed landmarks. Again, the animals were able to find their way after the partial, but not the complete removal, of the cues, suggesting that the entire spatial arrangement is embedded into a common reference framework as a unitary configuration. The tolerance to partial losses of spatial information has been proposed to be a key characteristic of the mammalian hippocampus-dependent map-like spatial memory [39,72,75,76]. This feature seems to depend on a pattern completion mechanism that is able to reinstate complete memories after partial cueing, based on the operation of hippocampal autoassociative neural networks [77,78,79,80,81].

**Figure 2:**
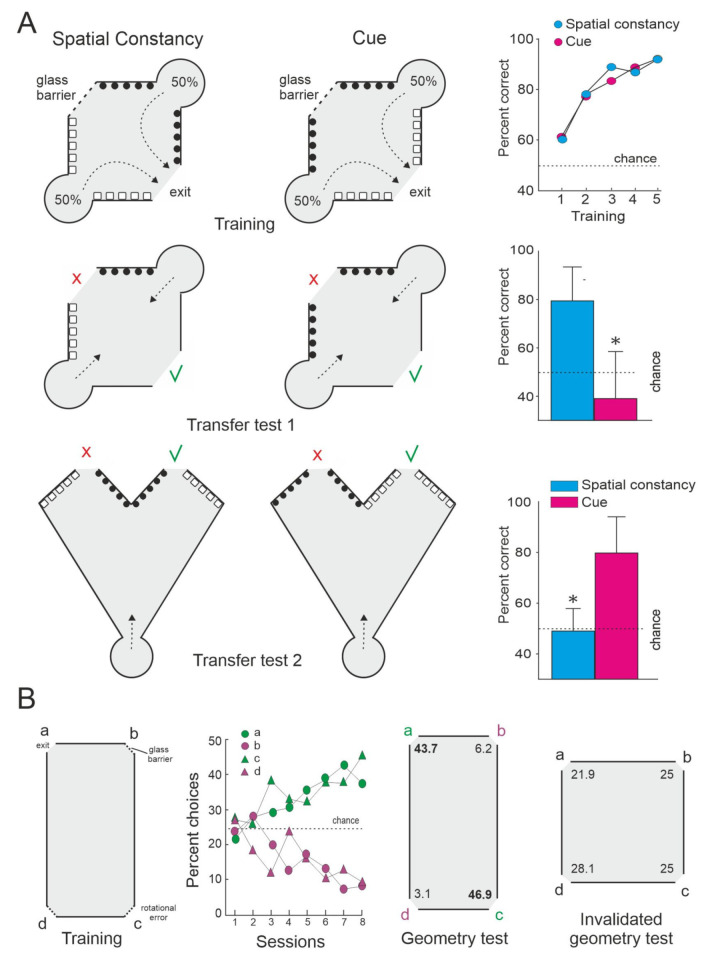
Relational map-like spatial representations in small stimulus-controlled mazes. (**A**) Two group of goldfish were trained to exit from an enclosure in a spatial constancy task which requires the use of allocentric (relational) strategies or in a cued version of the same task. The access from the start compartments, the distribution of the experimental visual cues (black and white symbols), the position of the glass barrier, and the location of the goal (exit) are shown for both training procedures. The numbers indicate the percentage of trials initiated from each start compartment. The arrows show the most efficient trajectories to the goal. Note that in the transfer tests the deletion of the local cues directly associated with the goal (Transfer Test 1) did not alter the performance in the relational task (spatial constancy), however the alteration of the global layout of the experimental setup (Transfer test 2) disrupted performance, even though the relationships between the local cues and the goal remained unaltered in the transfer tests. The green check marks indicate the door corresponding to the goal during training conditions. The figures on the right show the percentage of correct responses during training and transfer tests. Asterisks denote significant differences. Modified from [16]. (**B**) Encoding of geometrical spatial information by goldfish. Fish were trained to find the exit door (goal) placed in a corner (a) of a rectangular environment on the basis of the geometrical information provided by the apparatus. The arena had three identical, blocked openings (glass barriers) in the other three corners (b–d). Note that because of the geometric properties of the apparatus, the correct corner was indistinguishable from the diagonally opposite (180°) corner (rotational error). The percentage of choices for the four corners during training is shown. Two different probe trials were carried out in which the glass barriers were not used, so that fish could exit freely through any door. For the invalidated geometry test, a new apparatus that modified the geometric properties of the experimental enclosure was used. Numbers in the diagrams indicate the percentage of choices to each door during the tests. Modified from [87].

Additional laboratory studies support the idea that teleost fish can use relational map-like spatial representations. For example, López et al. [16] showed that whereas the deletion of an individual local visual cue directly associated with the goal was not detrimental for the performance of goldfish trained in a spatial constancy task (tasks solved by means of a spatial mapping strategy), the alteration of the global layout of the experimental setup (that modifies its whole geometry or the topological relationships between its constituent elements) dramatically disrupts performance, even though the relationship between the local cue and the goal remain unaltered (Figure 2A). The failure in the use of a strategy based on local cues after the massive modification of the global shape of the surrounding environment could be indicative of the triggering of a global remapping that lead the subjects to perceive the substantially altered experimental setup as a novel environment [74,80]. Interestingly, a number of studies have demonstrated also that teleost fish can rely on the geometry or global shape of the environmental boundaries and surfaces for orientation and navigation [24,82,83,84,85,86,87,88] (Figure 2B). Furthermore, like adult humans, monkeys, rats, and birds, teleost fish can use geometrical and non-geometrical information (i.e., the form or color of individual landmarks and surfaces), conjointly or alternatively depending on the task requirements [85,87]. In summary, these results suggest that teleost fish are able to encode different environmental elements (landmarks, relevant locations, and goals) and their spatial relationships (topographical and metrical information) in a common, unitary, map-like internal representation of the environment that provides a “world-centered” framework that enables allocentric navigation.

Furthermore, the goldfish in the Rodríguez et al. [17] study seem to be able to make spontaneous shortcuts and detours without previous route-specific experience, i.e., they accurately navigated towards the goal place, choosing the most direct trajectory to the goal during the transfer tests in which they departed from novel start locations, and even when the maze was displaced to new positions in the room (Figure 1E). The results suggest that these animals used a map-like spatial memory representation that endowed them with the capability to infer direct pathways to the goal place from unfamiliar start locations. It is important to mention that shortcutting and detouring behavior has been proposed as a key evidence to distinguish allocentric navigation based on cognitive maps or survey representations from more simple navigational strategies [17,32,39,50,51]. The cognitive map view proposes that the animals do not merely respond reflexively to the cue stimuli, but instead they acquire meaningful information about the spatial relationships in the environment, which enable them to make inferences (or to form “expectations”) about how places are connected through unknown terrains [39,50]. This inferential capability allows the animals to plan their paths and to undertake flexible and purposive navigational responses. A number of neurobiologically inspired, fully mechanistic computational models of navigational behavior have been developed to account for the ability of animals relying on allocentric frameworks to perform path planning, without the need to resort to “mentalistic” explanations (i.e., [51,89,90,91,92,93,94,95]. 

As a whole, these results indicate that teleost fish, like mammals [39,48,65,68,96], birds [97,98,99,100], and reptilians [101,102], can use allocentric strategies to solve spatial tasks. Interestingly, some laboratory studies in sharks and stingrays suggest that elasmobranchs may be also able to use allocentric spatial strategies based on some kind of map-like spatial memory [103,104,105]. Since elasmobranchs are considered a sister clade of actinopterygian fish, these results suggest the possibility that cognitive mapping is an ancestral navigational strategy that appeared early during vertebrate evolution.

## 2. Neural Mechanisms for Spatial Navigation

As it has been discussed in the previous section, teleost fish can navigate using a variety of sensory modalities and orientation strategies, from relatively simple egocentric mechanisms, to allocentric navigation based on hypothetical survey representations or “cognitive maps”. However, the terms “egocentric” and “allocentric” navigation frequently involve rather loosely defined concepts that may include an assortment of different cognitive and neural mechanisms under the umbrella of a common denomination. Thus, a complete understanding of the spatial navigation strategies used by teleost fish requires the identification of their separate brain substrata. A wealth of evidence shows that the diverse behaviorally dissociable spatial navigation strategies reviewed in the last sections can be separated also in terms of the brain centers and circuits that subserve them.

### 2.1. Neural Mechanisms for Egocentric Orientation in Teleost Fish

**Figure 3:**
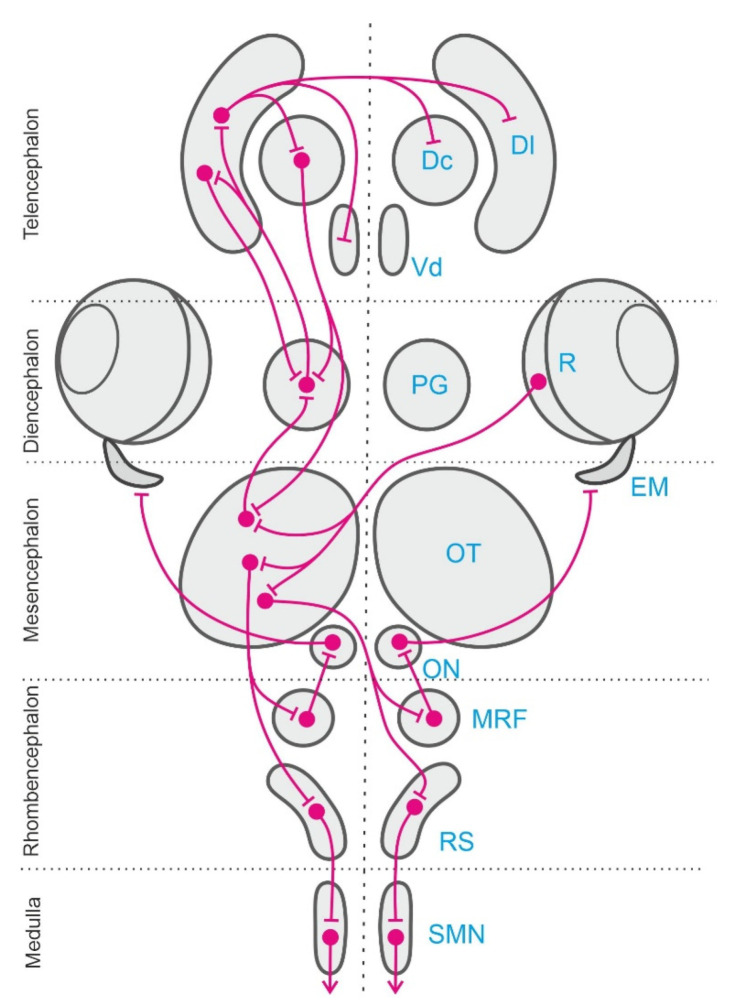
Schematic representation of the extended neural network involved in the egocentric sensorimotor transformations and egocentric-to-allocentric spatial reference framework conversion described in the text. Only the left half of the bilateral network is presented here. The right retina (R) sends visual information to the contralateral optic tectum (OT). Visual and other sensory modalities converge into the OT and in other stages of this neural network, where multisensory integration takes place. The multisensory information is represented in the OT in a body-centered map. Sensorimotor transformations leading to the generation of the egocentric orienting responses are performed at early processing stages in the OT, but also in parallel and sequentially in other nodes of the network. The tectal motor commands, encoded in egocentric coordinates, are conveyed to the neural circuits in the mesencephalic reticular formation (MRF) that organize the saccadic motor programs. These signals are submitted to further transformations into the OT-MRF interface to adapt to the specific requirements of the ocular motor plant, and in turn they finally activate the extraocular motoneurons in the oculomotor nuclei (ON) to produce orienting eye-movements (EM, extraocular muscles). Tectal efferences also activate reticulospinal (RS) assemblies, which in turn recruit spinal motor networks (SMN) to produce orienting (ipsilateral descending pathway) or avoidance (contralateral descending pathway) responses. In regard to the ascending (prosencephalic) tectal projections, the OT send massive efferents to the preglomerular complex (PG), the main diencephalic sensory relay station of teleost fish, which in turn project into the intricate telencephalic neural networks in which the egocentric-to-allocentric reference framework transformations are thought to take place. The main visual information recipient pallial region is the dorsolateral area (Dl), which has demonstrated to be a crucial center for allocentric navigation, with other sensory modalities reaching separate pallial targets. The telencephalic pallium sends back outputs to the PG, the OT, and other descending motor networks through the dorsocentral (Dc) pallial area and through other telencephalic subpallial structures, as the ventrodorsal nucleus (Vd) of the telencephalon, an area likely homologous to the tetrapod’s basal ganglia, which finally modulates behavioral responses.

Classical neurophysiological experiments and more recent studies using modern brain imaging and manipulation techniques have identified a number of brain centers and neural networks involved in egocentric orientation in teleost fish. These neural circuits include mainly several stages of the sensory and perceptual systems, some sensorimotor forebrain circuits, the optic tectum, the cerebellum, and various premotor centers and descending pathways in the brainstem and medulla [106,107,108,109,110,111,112] (see Figure 3). Several sensory systems and neural networks are involved in egocentric navigation in teleost fish. Some of these systems are shared with other vertebrates and seem to correspond to an ancestral design well-conserved in the different vertebrate radiations. This appears to be the case for the vestibular system, that provides a “sense of position” encoded in egocentric frames of reference anchored to the invariant direction of the gravity field and that also provide the sensory basis for inertial navigation [113,114,115,116], or for the optic tectum networks, that provide common body-centered frames of reference for multisensory integration and for sensory-motor transformations [112,117,118]. On the contrary, other systems are typical of fishes, for example the lateral line sensory system [23,24,25], or constitute notable examples of adaptive specializations, e.g., the electrosensory mechanisms that likely contribute to fish orientation [26,27,28,29,119].

A crucial brain center for the generation of egocentric orientation responses in fish is the optic tectum. This multilayered brainstem structure, homologue to the superior colliculus of mammals, reaches a remarkable degree of development in teleost fish. By virtue of its profuse connectivity with a variety of sensory and motor centers, and because of its characteristic intrinsic organization, the teleost optic tectum provides the basis for the multisensory integration and sensorimotor transformations required for a number of body-centered behaviors [108,110,111,112,117,120]. In teleost fish, like in mammals and other vertebrates such as birds, reptiles, and amphibians, the optic tectum is involved in the generation of orienting and avoidance responses of the incoming stimuli [112,121,122,123,124,125]. The optic tectum of teleosts receives sensory information mainly from multiple sensory modalities to form real-time representations of the animal surrounding, to identify significant stimuli, like preys, or potentially dangerous objects, as predators, and to trigger sensory guided responses. These responses include, for instance, food seeking and prey catching, orienting towards objects, schooling and swimming to maintain the position relative to the visual background, and avoidance of approaching objects and obstacles [110,126,127,128,129,130,131,132,133,134] Like in other vertebrates, the superficial layers of the teleostean optic tectum receive a topographically ordered primary retinal projection that forms a retinotopic visual map over the tectal surface [135,136,137]. The intermediate and deep layers of the optic tectum also receive spatially ordered inputs from other sensory modalities, such as auditory, somatosensory, and lateral line information, in topographical correspondence with the superficial retinotopic visual map [117,120,125,138]. The topographical overlapping of inputs from different sensory modalities is thought to be a mechanism that provides a common body-centered reference framework for multisensory integration [139,140]. In addition, the output neurons in the intermediate and deep layers of the teleost optic tectum are able to initiate orientation or avoidance responses through their projections to the reticular formation and other pre-motor cell groups in the brainstem, which in turn activate the motoneurons in the oculomotor nuclei and spinal cord that control orienting eye and body movements [107,109,110,111,128,141,142].

**Figure 4:**
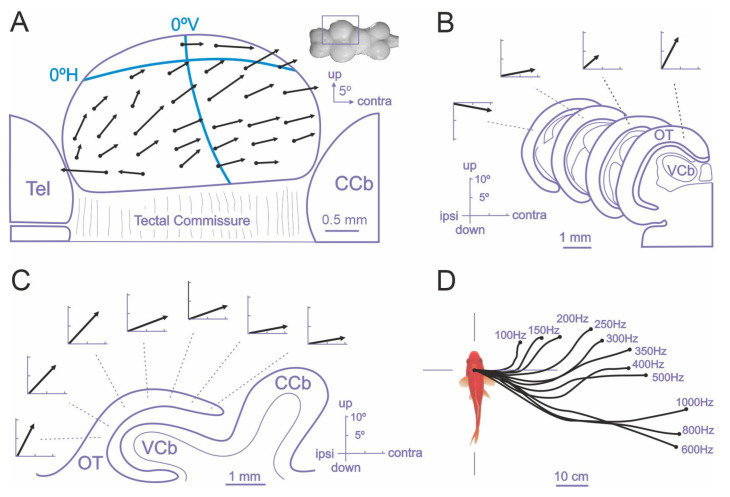
The optic tectum of teleost fish is a crucial brain center for the generation of egocentric orientation responses. Electrical microstimulation in the optic tectum of goldfish elicits coordinated eye (**A**–**C**) and body (**D**) movements. (**A**) Vectorial representation (black arrows) of the evoked eye movements after focal electrical stimulation in the optic tectum, showing the amplitude and direction of the saccades. Note that the amplitude and direction of eye movement vectors depend on the stimulation site within the tectum. The retinotopic vertical and horizontal axis are superimposed (blue lines). (**B**) Variation of the stimulation site in the medial–lateral axis produces an increase in the vertical component. (**C**) Variation in the stimulation sites across the rostro–caudal axis produced a systematic change in the amplitude of the horizontal component of the saccade. (**D**) The direction and amplitude of the orienting responses in free-swimming fish depend on both the tectal stimulation site and the stimulus parameters. The insert in (**A**) shows a dorsal view of the goldfish brain and the square marks the magnified area. CCb: corpus cerebellum; OT: optic tectum; Tel: telencephalon; VCb: valvula cerebellum; ipsi and contra: ipsiversive and contraversive direction of evoked eye saccade, respectively. Modified from [131,133].

The role of the teleost optic tectum in sensorimotor transformations and in the generation of orientation responses has been thoroughly studied in goldfish by focal electrical microstimulation experiments [131,132,133] and more recently in the larval zebrafish using neuroimaging and optogenetic techniques [108,110,127,128,130,134]. Electrical stimulation of the intermediate and deep layers of the optic tectum of goldfish triggers orienting responses consisting of coordinated eye and fin movements, axial musculature adjustments, and locomotion behavior that closely resemble natural orienting responses [126,127,131,132,133,143,144]. The direction and amplitude of the evoked orientation responses varied systematically with electrode position within the tectal lobe extend, indicating the presence of a spatially ordered motor map in the deep tectal layers, roughly in correspondence with the retinotopic visual map of the superficial layers, and suggesting that the tectal evoked orientation responses are encoded in a body-centered reference framework [131,132,133]. In particular, the careful measurement of the gaze shifts evoked by focal electrical microstimulation of the intermediate and deep layers of the optic tectum of restrained goldfish revealed the presence of a “vector map” of eye movements [133] (Figure 4). The “characteristic vector”, i.e., the amplitude and direction, of evoked eye movements depended on the stimulation site within the tectal lobe. The displacement of the electrode position in the rostro-caudal direction produced a systematic increment in the amplitude of the horizontal component of the eye movement, whereas the displacement of the stimulation site in the medial-lateral axis produced an increase in the vertical component, revealing a topographically ordered motor map (Figure 4A–C). The motor map found in the deeper layers of the optic tectum was roughly aligned with the retinotopic visual map present in the superficial tectal layers, as it seemed to direct gaze to the corresponding points in the retinotectal map (the upper visual field being represented medially in the tectal lobe and the caudal visual field caudally, Figure 4A). Thus, these results suggest that the tectum encodes gaze orientation responses by means of a place code specified by the topography of the tectal motor map, that determines the direction and amplitude of the fish eye movements, although also the amount of activity in a given tectal loci can influence some characteristics of the evoked saccades, i.e., by means of a population or activity code [133] (Figure 4D). Further, the spatially coded tectal commands must be transformed into a temporal signal that specifies the required force and duration of extraocular muscle contractions into the tecto-reticular-oculomotor interfaces (Figure 3). These spatial-to-temporal code transformations are thought to depend on the anatomical organization of the tecto-reticular projections to the separate brainstem generators for horizontal and vertical eye movements [110,111,145,146,147].

Thus, an extensive network of sensory-to-motor interfaces operates in the teleost fish brains to acquire and process spatial information encoded in egocentric coordinates and to generate actions in the space using body-centered frames of reference. The teleostean optic tectum can be considered an important neural hub of this brain network that provides a common body-centered frame of reference for multisensory integration and sensory-motor transformations, being a crucial center for the generation of egocentrically referenced actions in space [53,133]. In addition, other brain centers and neural systems in the teleost fish brain, for example, the hippocampal telencephalic pallium and related structures (Figure 3), are thought to be specialized to transform the egocentric information into “world-centered” spatial frameworks to construct allocentric representations of the environment that are independent of the subject’s own view or position within it [53,148].

### 2.2. Teleost Fish Hippocampal Pallium and Map-Like Navigation

A considerable amount of evidence shows that the medial pallium or hippocampus of land vertebrates plays a central role in allocentric spatial navigation. For example, in mammals [39,57,72,149], birds [97,98,99,150,151,152,153], and reptiles [112,154,155,156,157], the hippocampal lesions produce impairments on spatial tasks that require the use of map-like or relational spatial memory. The hippocampus of tetrapods seems to be essential in tasks requiring the formation of a memory of the spatial relationships of multiple elements and cues of the environment and those demanding the flexible expression of previously acquired spatial knowledge [72,73,75,76]. Interestingly, comparative neurobiological research shows that the telencephalon of ray-finned fishes (actinopterygians) can also contain a region homologue to the hippocampus of tetrapods [158,159]. The telencephalon of actinopterygian fish presents a very divergent process of development compared with all other vertebrates, namely the “folding out” or eversion of the embryonic telencephalic walls, that contrast with the “folding in” or evagination that takes place in non-actinopterygian vertebrates [160,161,162,163]. Nevertheless, despite this striking difference, the telencephalic pallium of actinopterygians seems to share at least some of its basic divisions with the pallium of non-actinopterygians, even though the topography of the different pallial regions is thought to be roughly reversed compared with the pallium of all other vertebrates as a consequence of the process of eversion [159,161,162,164,165]. Thus, considerable agreement exists that the telencephalic pallium of teleost fish contains regions homologous to the mammalian hippocampus, olfactory cortex, pallial amygdala, and dorsal cortex (although this last aspect is still disputed) [112,164,165,166,167,168,169].

Taking into account the process of telencephalic eversion in teleost fish and the fact that the hippocampus originates from the most distal region of the embryonic prosencephalic alar plate in land vertebrates, the pallial area considered homologous to the hippocampus in teleost fish is the dorsolateral telencephalic region [158,159,170,171,172,173]. The presumed homologue of the hippocampus in teleost fish shares not only a comparable topological position within the pallium, but also a number of developmental, neuroanatomical, connectivity, and histochemical similarities. Thus, the cells of the teleost fish dorsolateral telencephalon have an embryonic origin corresponding with a conserved “hippocampal” topological identity [174]. In addition, developmental gene studies showed the expression of highly conserved gene “markers” of the mammalian and avian hippocampus and dentate gyrus in the putative teleost hippocampal pallium [166,175]. Like the hippocampus of land vertebrates, the presumed hippocampal pallium of teleost fish receives diencephalic inputs from different sensory modalities, present widespread reciprocal connections with other pallial areas, is reciprocally connected with a cholinergic area considered homologous to the septal nucleus of tetrapods as well as with the preoptic area, and receives inputs from the locus coeruleus and the superior raphe [164,167,176,177,178,179]. This pattern of connectivity is reminiscent of that of the hippocampus of amniotes.

Noteworthily, the putative hippocampal pallium of teleost fish, like the hippocampus of land vertebrates, is also a pivotal brain center for map-like, allocentric spatial navigation. A growing number of experimental studies using well-controlled behavioral procedures combined with brain lesion, neural activity recording, and neuro-morphofunctional techniques have provided evidence on the critical role of the teleost fish hippocampal pallium in spatial cognition [28,112,157,180,181,182,183,184,185,186]. For example, it has been reported that spatial learning produced selective increases in protein synthesis [181,185] and in metabolic activity [183,184] in the dorsolateral telencephalon of goldfish, and significant increases in the mRNA levels of the immediate early genes *bdnf* and *egr-1* in the dorsolateral telencephalon of the cichlid *Astatotilapia burtoni* [187]. In addition, dorsolateral telencephalon lesions in teleost fish produced significant deficits in spatial navigation tasks that required the use of mapping capabilities and allocentric strategies, but not in tasks that could be solved by egocentric strategies or non-spatial discriminations [157,181,182].

**Figure 5:**
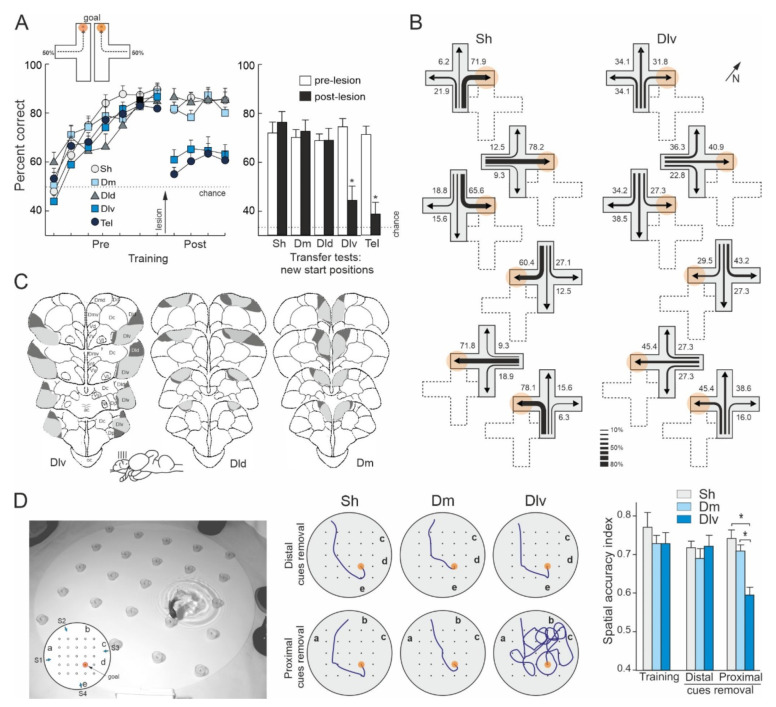
Dorsolateral telencephalon lesions in teleost fish produce significant deficits in spatial navigation tasks that require the use of map-like strategies. (**A**) Effects of different pallial lesions on the learning of a place task in a plus maze (see Figure 1). The curves show the mean percentage of correct choices during pre- and post-lesion training sessions. The histograms show the percentage of correct choices during the transfer trials in which new start positions were employed. Asterisks denote significant differences. (**B**) Trajectories chosen by sham-operated (Sh) and dorsolateral telencephalon-lesioned (Dlv) goldfish during the transfer trials conducted after surgery, in which the maze was displaced, and new start positions were employed. The numbers and the relative thickness of the arrows denote the percentage of times that a particular choice was made. The position of the maze during training trials is shown by dotted lines. Note that the Sh goldfish consistently chose the route leading to the place where they were rewarded during the training trials (orange circle). In contrast, the random distribution of the choices by Dlv-lesioned animals revealed a profound spatial deficit. (**C**) Schematic transversal drawings of the telencephalon of goldfish showing the largest (dark grey) and the smallest (light grey) extensions of the different pallial lesions. Dm: dorsomedial telencephalon-lesioned group; tel: telencephalon ablated group. Modified from [157]. (**D**) Effects of dorsomedial (Dm) and dorsolateral (Dl) telencephalon lesions in the use of allocentric strategies to locate a goal in a hole-board homologue task. At the right is shown a photograph of the experimental apparatus and the training procedure. In the insert is shown the goal (baited feeder, red circle), the position of the cues (letters a–e), and the four different start positions used during training (S1–S4). The diagrams on the right show the searching trajectories of a representative fish of each group on the distal and proximal cue-removal tests. Note the spatial deficit of Dlv goldfish when the cues in the vicinity of the goal were removed. The histogram shows the mean spatial accuracy index (values relative to distance to the goal) in training and in the probe tests in which the distal (a,b) or the proximal (d,e) cues to the goal were removed. Asterisks denote significant differences. Modified from [182].

Rodríguez et al. [157] trained goldfish to solve a place task in a plus-maze surrounded by widely distributed distal visual cues. After mastering the task, the animals were submitted to lesions to the dorsolateral, dorsodorsal, or dorsomedial regions of the telencephalon. Remarkably, only the goldfish with lesions in the dorsolateral telencephalon showed a profound spatial navigation impairment, as their performance decreased nearly to chance after surgery, being unable to find the previously learned goal location (Figure 5A). In addition, the dorsolateral telencephalon-lesioned goldfish were unable to find the goal location during the postsurgery transfer tests in which they were forced to depart from new start places (shortcutting impairment, Figure 5A,B). Moreover, the dorsolateral telencephalon lesions produced an impairment as severe as the ablation of the complete telencephalon (see also [71]). In contrast, no spatial deficits were observed in the goldfish with lesions in the dorsodorsal or the dorsomedial telencephalon, as they maintained the same level of proficiency that before the lesion, and similar to that of the animals in the sham group, even when reaching the goal implied using novel routes when they departed from new start places during the transfer tests (Figure 5A,B). Furthermore, the deficit observed in the dorsolateral telencephalon-lesioned goldfish seems to be highly selective for spatial navigation and place-memory, as the damage to this area did not impair performance in a cue-learning task in which the goal was directly signaled by a conspicuous intramaze cue. Thus, like the hippocampal pallium of reptiles, birds, and mammals, the dorsolateral telencephalon of teleost fish seems to be essential for map-like based allocentric navigation and for shortcutting behavior, but not for simple cue-learning and non-spatial discriminations. As a whole, these results demonstrated a striking functional similarity between the dorsolateral area of the telencephalon of teleost fish and the hippocampal pallium of land vertebrates.

Durán et al., [182], using different behavioral procedures, obtained convergent results regarding the involvement of the goldfish presumed hippocampal pallium homologue in map-like spatial navigation. In this study, goldfish with lesions in the dorsolateral or in the dorsomedial telencephalon were trained in a place-learning task in a well-controlled spatial laboratory environment (Figure 5D). The animals were required to learn the location of a baited feeder within a square matrix of 25 feeders distributed in a large circular aquarium. The feeder matrix was surrounded by a peripherally distributed array of five landmarks. The baited feeder maintained a stable spatial relationship relative to the array of landmarks during the training trials. Four different start positions were randomly used along the training in order to potentiate place-strategies to solve the task. With training, the number of errors decreased significantly in all lesion groups and the goldfish learnt to locate the baited feeder with accuracy, employing direct trajectories to the goal. Remarkably, although between-group differences were not observed in the ability to find the baited feeder during the training trials, the results of the probe tests indicated that the performance of the animals in each lesion group was based on very different spatial strategies. The goldfish with dorsomedial telencephalon lesions, like the sham-operated animals, accurately navigated to the goal place despite the partial removal of the peripheral landmarks (proximal- and distal-cue removal tests) and even when each one of the landmarks was individually removed (single cue-removal tests; Figure 5D). In fact, their performance only declined when the complete array of landmarks was removed or massively transposed. These results indicated that the dorsomedial-lesioned and sham-operated goldfish relied on the peripheral landmarks for navigation, but, notably, none of the cues was essential by itself to locate the goal. Thus, the results of the probe tests suggest that these animals used a map-like, relational spatial memory representation of the experimental environment that allowed them to flexibly and reliably navigate to the goal regardless of the removal of some of their constituent elements. In contrast, although the performance of the dorsolateral telencephalon-lesioned goldfish did not differ from that of the animals in the other groups during training trials, they dramatically failed in the probe tests in which the landmarks in close proximity to the baited feeder were removed (Figure 5D). These results demonstrated that the dorsolateral telencephalon-lesioned goldfish relied exclusively on the individual local cues in the immediate vicinity of the goal place to solve the task, and not on the complete array of landmarks. Thus, the results of the experiment of Durán et al. [182] showed that whereas the dorsomedial telencephalon-lesioned and the sham-operated goldfish were able to use a navigation strategy based in a map-like relational representation of the environment that included the spatial relationships of the goal place with the arrangement of landmarks as a whole, the dorsolateral telencephalon-lesioned goldfish suffered a profound spatial cognition deficit, as they only could solve the task using a guidance strategy consisting in approaching a particular subset of cues. These results closely resemble those obtained after hippocampus lesions in mammals, birds, and reptiles [39,57,72,97,98,112,152,157] and demonstrated that the dorsolateral telencephalon of teleost fish is a critical brain center for allocentric navigation based in map-like spatial memory.

## 3. Hippocampal Pallium Mechanisms for Map-Like Spatial Navigation in Teleost Fish

As discussed in the precedent section, the teleost fish hippocampal pallium, like the hippocampus of land vertebrates, seems to be essential for navigation based on map-like spatial memory. Recent neurobiological research has identified some of the hippocampal mechanisms that likely support map-like spatial memory and allocentric navigation in teleost fish. In the next three subsections we will review some of this evidence, including (i) single-unit recording data on cells with spatial-related activity in the telencephalic pallium; (ii) some recent evidence on the dynamic processes of spatial memory encoding and retrieval; and (iii) finally, we will discuss the possibility that spatial memory in teleost fish should be merely considered a special case of a more wide relational memory system that encodes both the spatial and the temporal dimensions of episodic-like memories.

### 3.1. Space-Related Cells in the Pallium of Teleost Fish

In mammals, allocentric navigation is thought to involve the operation of an extended cortico-hippocampal network that conveys highly processed visual and multisensory information to the hippocampal circuits for further processing and transformation into “world-centered” relational spatial representations [74,76,79,188,189,190,191,192]. Mammalian extra-hippocampal cortical areas contain a variety of functionally specialized neurons with presumably distinct roles in the representation of space, i.e., “head direction cells”, “border cells”, and “grid cells”, that likely encode relevant spatial information in egocentric coordinates [72,193,194,195,196]. The egocentric spatial information coded by these neurons is thought to be further transformed into an allocentric representation downstream in the cortico-hippocampal network. The “place cells”, i.e., hippocampal neurons that fire when the animal occupies a particular location of the space, have been proposed to be the higher instance of a brain system that builds up map-like spatial memory representations anchored in the external world, contributing to the neural computations required for allocentric navigation [39,72,74,197,198].

Although the neuroanatomy and neurophysiology of the teleost fish telencephalon is not completely understood, recent comparative neurobiological research has provided interesting insights on the functional organization of the pallial telencephalic network essential for map-like spatial memory in this vertebrate group. The pallium of teleost fish receives information from all sensory modalities, from the preglomerular complex and the thalamus, the main ascending sensory diencephalic relay stations in teleosts [164,165,169]. These modality-specific sensory projections reach separate areas of the pallium, and also overlap in “association” pallial areas [169,178,179,199,200,201]. Finally, like the hippocampus of mammals and other tetrapods, the teleost fish hippocampus homologue is profusely interconnected with these primary and association sensory pallial areas [164,175,199] from which it seems to receive highly processed sensory information required to construct map-like spatial memories and for allocentric navigation [175,183]. Interestingly, single-unit recording studies in free swimming fish have revealed the presence of neurons in the telencephalic pallium of teleosts whose firing rate seems to encode spatial navigation-related features. Thus, Canfield and Mizumori [202] reported the presence of cells in the dorsolateral telencephalon of cichlids and goldfish that appear to display some location-specific discharge. Takahashi et al. [203] described directionally tuned cells in the telencephalic pallium of rainbow trout that fired when the fish head was oriented in a specific direction. In a more comprehensive study, Vinepinsky et al. [186] likewise showed the presence of cells with navigation-related activity in the dorsolateral telencephalon of free-swimming goldfish, for example, “border cells”, that increase their firing rate when the fish approaches the environmental boundaries, and “velocity cells”, whose activity correlate with the fish swimming direction and speed. Additionally, Fotowat et al. [28] and Trinh et al. [204] found cells in the hippocampal pallium of an electrosensory gymnotiform that presumably exhibit spatial navigation-related activity. Furthermore, recently Wallach et al. [205], based on electrophysiological single cell recordings in the preglomerular complex of the weakly electric fish *Apteronotus leptorhynchus*, have proposed a hypothetical neural mechanism to derive allocentric spatial representations from egocentric sensory information. As the neurons in the preglomerular complex precisely encode the time-interval between object encounters during spatial behavior, Wallach et al. propose that this temporal information, combined with a fish locomotion speed signal, can allow the accurate estimation of the distance between object encounters, contributing both to path integration-based navigation and to computing allocentric spatial relationships in the environment. These spatial-coding cells in the telencephalic pallium of teleosts and related brain areas may be part of a neural network that likely represents the spatial structure of the environment, including the geometric configuration of borders and landmarks relative to the subject, as well as some dynamic parameters relevant for navigation, such as velocity, direction, and relative position with regard to surfaces and objects. Such a neural network may provide the basis for map-like spatial memory representations that can support the ability for allocentric navigation in teleost fish.

### 3.2. Spatial Memory Encoding and Retrieval in Teleost Fish

Map-like spatial memory representations could endow teleost fish spatial behavior with considerable flexibility, providing the capability to infer new spatial relationships during shortcutting and detouring behavior, to rapid learn the new goal location during spatial reversal tasks, or to resist partial losses of spatial information, e.g., following the partial removal of subsets of relevant landmarks. It has been proposed [79,80,190,206,207] that similar spatial behavior capabilities likely depend, in mammals, on two hypothetical hippocampal mechanisms: first, the projections from the dentate gyrus to the CA3 area of the hippocampus seem to operate as a sparse coding device that orthogonalizes and disambiguates similar inputs patterns, performing pattern separation during initial encoding and memory storage; second, the extensive recurrent-collateral network of the CA3 neurons operates as an autoassociative network that allows the storage of arbitrary associations between stimuli, and that functions as a pattern completion mechanism that reinstates the complete memories in response to incomplete input patterns. Thus, according to the mechanistic models of hippocampal memory function, the hippocampal neural network can shift between two functional modes, encoding and retrieval, depending on the novelty degree of the incoming spatial information [77,78,79]. At the initial stages of learning, when the animals confront novel spatial information, the hippocampal network engages in a storage mode, characterized by pattern separation and encoding memory operations. Later, when learning progresses and the novelty of the incoming information decays, the hippocampal network spontaneously shifts to a recall mode, characterized by pattern completion and retrieval memory operations. Therefore, these models propose that spatial memory is a dynamic process in which different hippocampal circuits and mechanisms sequentially engage in a time-dependent manner throughout the different phases of memory formation and remembering [208,209,210,211,212,213]. Interestingly, recent neuroanatomical and neurofunctional studies have evidenced the presence of both sparse input projections and highly recursive intrahippocampal circuits in the presumed teleost fish hippocampal pallium homologue [214,215], as well as dynamic changes in the subregional hippocampal activity throughout the spatial learning process, suggesting pattern separation and completion operations that resemble those accomplished by the mammalian dentate gyrus/CA3 network [183,204]. The activation and engagement of distinct components of the hippocampal pallium at separate stages of spatial memory formation and recall have been recently reported in goldfish [183]. Using quantitative cytochrome oxidase histochemistry, Ocaña et al. [183] analyzed subregional progressive changes in the metabolic activity of the dorsolateral telencephalon of goldfish at different time-points during the learning of a spatial task. Interestingly, the activity level of the most rostral area of the ventral part of the dorsolateral region of the telencephalon (Dlv) increased significantly at the beginning of training, and progressively decreased as performance reached an asymptotic level, returning to pretraining values when the animals mastered the task. This evolving activity profile is suggestive of a specific role of this area in pattern separation and encoding computations during the initial stages of spatial memory formation, resembling the operation of the mammalian dentate gyrus/CA3 network. In contrast, the posterior area of Dlv, like the mammalian CA3 field, showed sustained activation across the whole training period, not only during the acquisition trials, when the pattern separation and encoding operations are thought to be dominant, but also after the animals mastered the task, and supposedly, the prevailing memory operations were pattern completion and retrieval. Thus, this time evolving pattern of metabolic activation appears to be indicative of the sequential engagement of separate subregions of the goldfish hippocampus in particular network computations that are typical of storage versus retrieval memory operations. These results suggest the possibility that map-like spatial memory could share some essential neural mechanisms in both land vertebrates and teleost fish.

### 3.3. Hippocampal Map-Like Memory in Teleost Fish: More Than Space?

Increasing amounts of evidence suggest that the hippocampal pallium of teleost fish, like the hippocampus of mammals, birds, reptiles, and amphibians, is involved in map-like spatial memory, supporting allocentric navigation. However, the function of the hippocampus of mammals is not limited to spatial navigation, as it seems to have a broader role in episodic memory. Thus, the mammalian hippocampus seems to be also essential to encode the time and order of events, to associate temporally separate stimuli, and to register non-spatial contextual information [216,217,218,219]. Interestingly, some experiments suggest that the dorsolateral telencephalon of teleost fish, like the mammalian hippocampus, is also involved in non-spatial relational memories. For example, hippocampal pallium-lesioned goldfish severely impairs the acquisition and retention of a trace two-way active avoidance conditioning task, but not of a delay version of the same task [220]. In the trace avoidance task, a stimulus-free temporal gap separated the warning conditioned stimulus (CS, a light) from the unconditioned stimulus (US, a mild electric shock), whereas in the delay avoidance task the CS and the US overlapped in time. These results, that closely resemble those obtained in hippocampal-lesioned mammals [221,222,223,224], suggest a critical role of the goldfish dorsolateral telencephalon in encoding the temporal dimension of associative memories. More recently, Rodríguez-Expósito et al. [225] provided additional evidence on a possible role of the teleost fish hippocampal pallium in the association of temporally separate events that compose episodic-like memories. In this study goldfish with dorsolateral telencephalic lesions, telencephalon ablation, or sham operation, were trained in a trace classical conditioning procedure in which the CS and the US were separated by a stimulus-free interval, in a delay procedure in which both stimuli overlapped in time, or in a long-delay conditioning procedure matched for trace duration. Remarkably, dorsolateral telencephalon lesions dramatically impaired acquisition of the trace conditioning task but spared both short- and long-delay conditioning. The impairments produced by the dorsolateral telencephalon lesions were as severe as those produced by the complete telencephalon ablation. These deficits are strikingly similar to those reported in hippocampal-lesioned mammals [216,226,227,228,229,230,231], and reveal that the hippocampal pallium of teleost fish is critical for the temporal dimension of the associative memories required for trace classical conditioning. Thus, these results could indicate that the hippocampal pallium of teleost fish, like the hippocampus of mammals, evolved as a neural device that endows the animals with the notable capability to navigate through the spatial and temporal dimensions of relational memories, suggesting that at least some of the basic features of the hippocampal-dependent episodic-like memory system may have appeared very early in the evolution of vertebrates.

## 4. Conclusions

Fishes have been traditionally thought to be “primitive” vertebrates that possess diminished behavioral and cognitive capabilities compared to land vertebrates. This belief has been sometimes sustained by the scarcity or even the total absence of dedicated studies. In fact, fishes are extremely successful and diverse vertebrates in terms of morphology, physiology, and behavior, and increasing evidence shows that at least some fish species have complex brains and are capable of sophisticated behaviors. This may be the case for teleosts, probably the most intensively studied fish group. In particular, a number of studies have analyzed in some depth their spatial navigation behavior and neural substrata, showing that teleosts use advanced navigational capabilities that closely parallel those of mammals and birds. Teleost fish rely on a multiplicity of sensory systems and can use a variety of spatial strategies for navigation. These strategies range from egocentric orientation to the use of allocentric map-like memory representations of the environment. Separate brain mechanisms subserve these distinct spatial strategies in teleost fish. One important hub for egocentric orientation, among others, is the optic tectum and associated brain circuits, that provide the basis for body-centered sensory integration and sensorimotor transformations involved in the generation of egocentrically referenced actions in space. An increasing number of studies indicate also that the dorsolateral telencephalic pallium of teleost fish, a brain region likely homologue to the hippocampus of land vertebrates, is a crucial center for allocentric navigation based on map-like spatial memory. Recent relevant studies have provided significant advancement in the understanding of the neural mechanisms of relational spatial navigation. Regrettably, information on other fish taxa, such as agnathans, cartilaginous fishes, and lungfishes, are extremely scarce. Behavioral and neurobiological comparative information on these fish taxa is however essential to draw a complete picture of the spatial cognition capabilities and its phylogenetic evolution in vertebrates. Nowadays there is a marked increase in attraction for fish models in neurobiology, as well as a general renewal of interest in evolutionary neuroscience, that hopefully will prompt comparative studies that could fill this gap.
